# Supplementary material for: In Vitro Efficacy and Molecular Mechanism of Curcumin Analog in Pathological Regulation of Spinocerebellar Ataxia Type 3
Source: Antioxidants (Basel). 2022 Jul 18;11(7):1389. doi: 10.3390/antiox11071389 (PMC9311745; doi:10.3390/antiox11071389)
Supplement: Supplementary file 1 [file antioxidants-11-01389-s001.zip › antioxidants-1771369-SI.pdf]

antioxidants

Supplementary Material

Efficacy and Molecular Mechanism of Curcumin Analog in Pathological Regulation of Spinocerebellar Ataxia Type 3 In Vitro

Yu-Ling Wu [1,†], Jui-Chih Chang [2,3,†], Yi-Chun Chao [4], Hardy Chan [5], Mingli Hsieh [6] and Chin-San Liu [1,7,8,9,*]

Supplementary Methods

Analysis of mitochondrial morphology

Imaging of mitochondria was performed to investigate whether JM17 alters mitochondria morphology (Chang et al., 2016). The effect of JM17 on MJD78 cells was monitored by confocal microscopy in dynamic manners (Figure S3). For visualization of mitochondria, cells treatment after 24 h were seeding in a chamber slide (μ -Slide 8 well, Ibidi GmbH, Munich, Germany) and stained with Mitotracker Deep Red (100 nM) (Invitrogen, Carlsbad, CA, USA) for 40 min. Labeled cells were removed remained dye and imaged in culture media at 37°C using an Olympus FluoView FV 1200 Confocal Microscope (Olympus, Tokyo, Japan).

Supplementary Figures

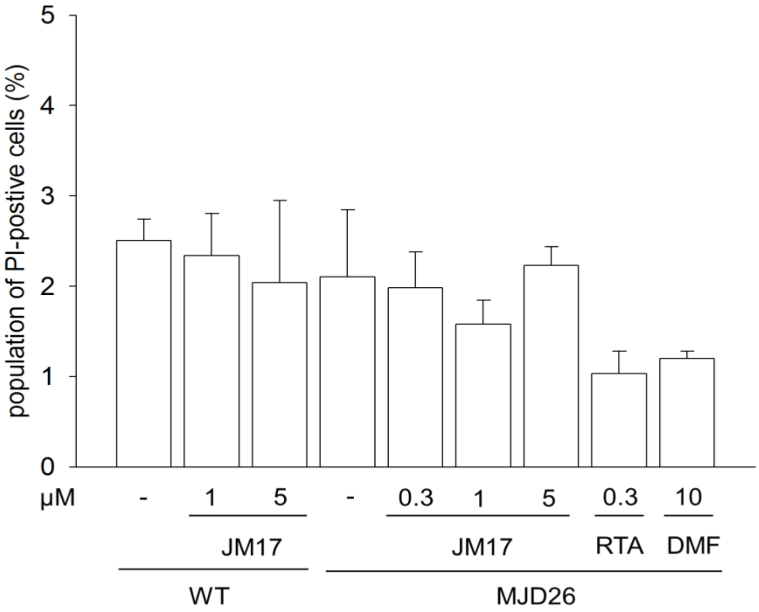

Figure S1. Effects of JM17, RTA-408, and DMF on cell viability in WT and MJD26 cells. WT cells were treated with or without DMSO vehicle control or with 1 or 5 μM JM17 and MJD26 cells were treated with or without DMSO vehicle control or with 0.3, 1, or 5 μM JM17, 0.3 μM RTA-408, or 10 μM DMF for 24 h. Cell viability was assessed by flow cytometry analysis using propidium iodide to stain dead cells. Data are presented as the mean ± SD of at least three independent experiments.

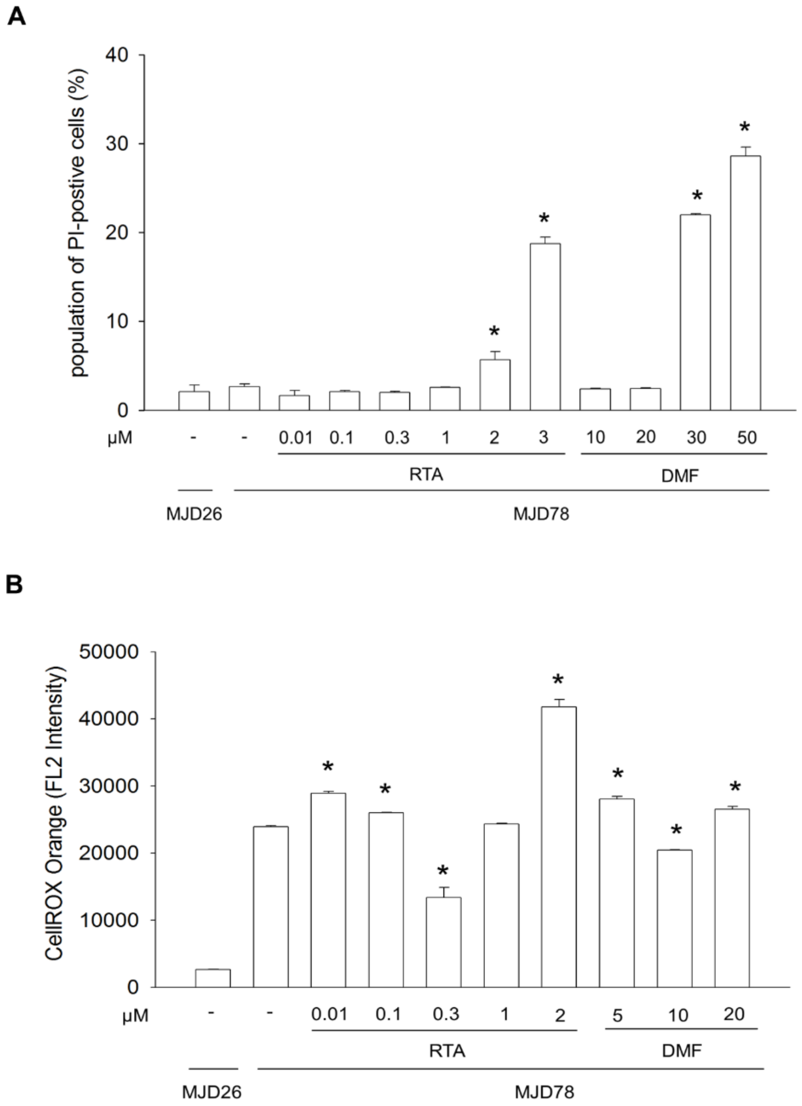

Figure S2. Effects of RTA-408 and DMF on cell viability and total ROS levels in MJD78 cells. (A) Cells were treated with or without DMSO vehicle control and treatment with 0.01, 0.1, 0.3, 1, 2 and 3 μM RTA-408 or 10, 20, 30 and 50 μM DMF for 24 hours. Cell viability was assessed by flow cytometry analysis using propidium iodide (PI) to stain dead cells. (B) Cells were treated with or without DMSO vehicle control and treatment with 0.01, 0.1, 0.3, 1 and 2 μM RTA-408 or 5, 10 and 20 μM DMF for 24 hours. Total ROS was measured by CellROX Orange staining and was quantified by flow cytometry. Data are presented as the mean ± SD of at least three independent experiments. Values from the treated cells were normalized to those of the MJD78 cells treated with vehicle only. *$p < 0.05$, compare to non-treated MJD78 group.

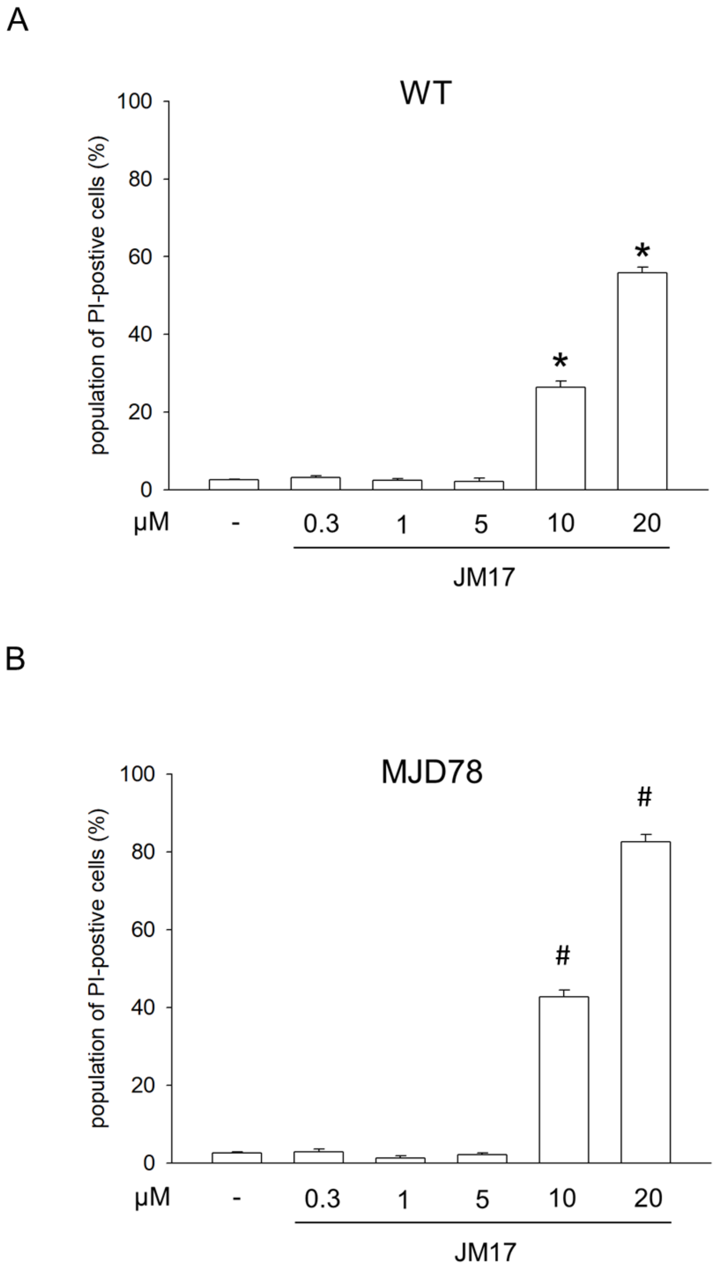

Figure S3. Effects of JM17 on cell viability in WT and MJD78 cells. (A) WT and (B) MJD78 cells were treated with or without DMSO vehicle control or with 0.3, 1, 5, 10 and 20 μM JM17 for 24 hours. Cell viability was assessed by flow cytometry analysis using propidium iodide (PI) to stain dead cells. Data are presented as the mean ± SD of at least three independent experiments. Values from the treated cells were normalized to those of the vehicle only. *$p < 0.05$, compare to non-treated WT group; #$p < 0.05$, compare to non-treated MJD78 group.

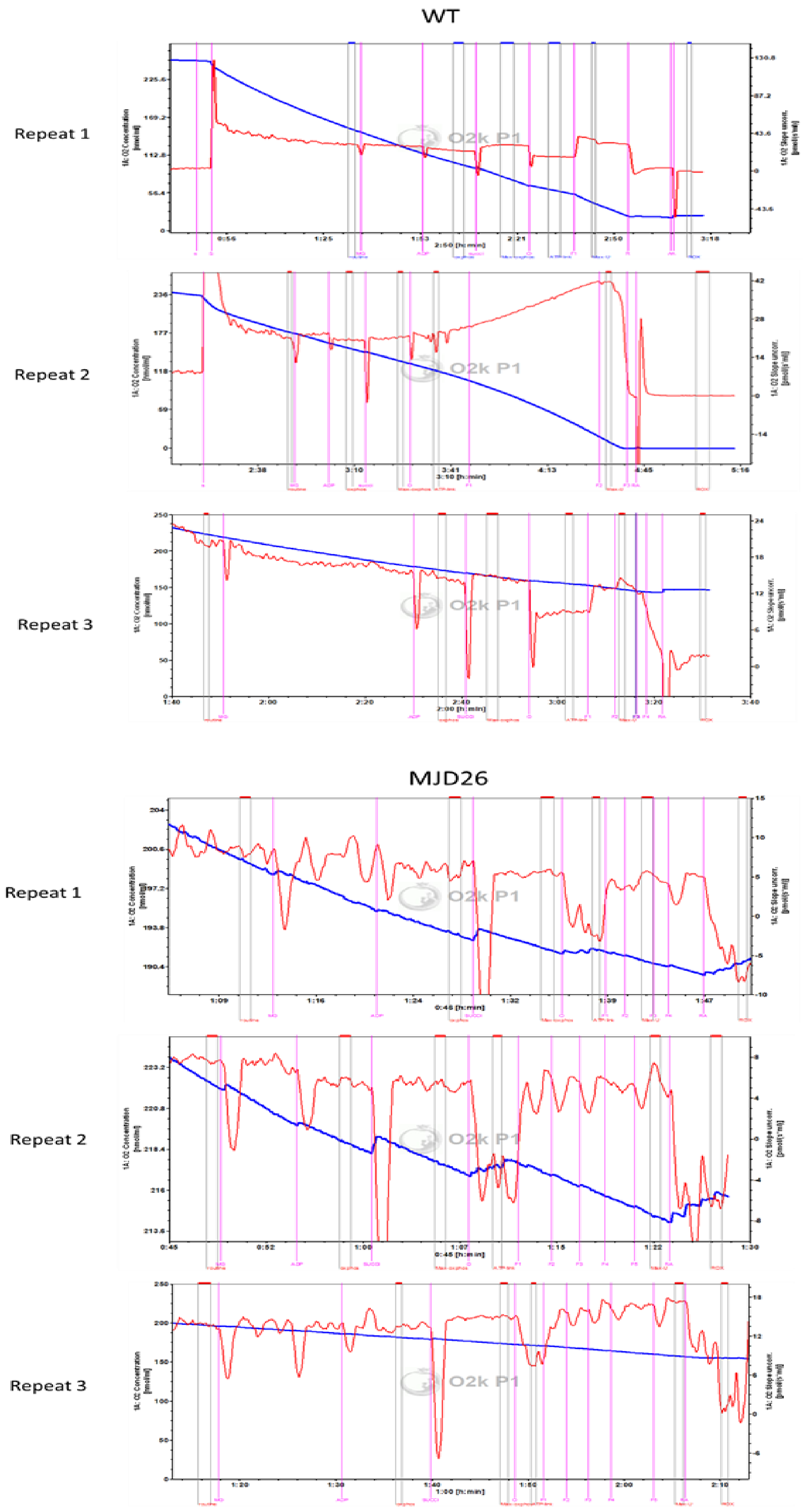
WT
Repeat 1
Repeat 2
Repeat 3
MJD26
Repeat 1
Repeat 2
Repeat 3

MJD78 DMSO

Repeat 1

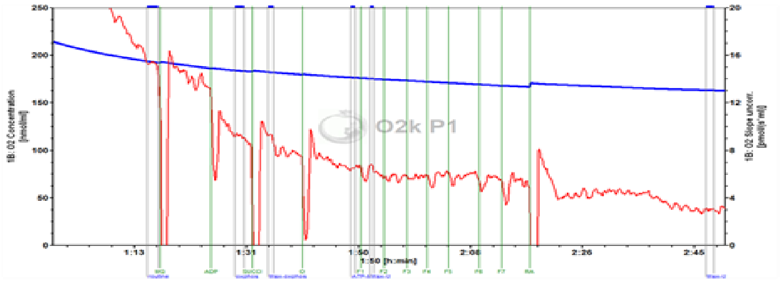

Repeat 2

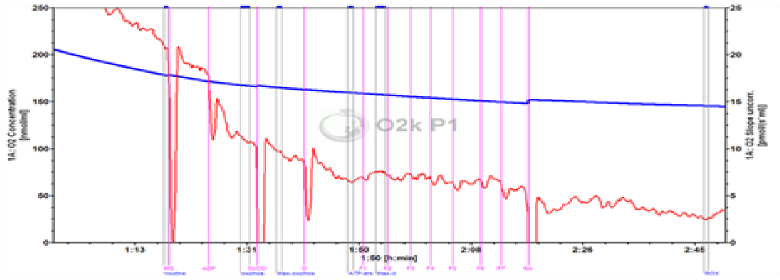

Repeat 3

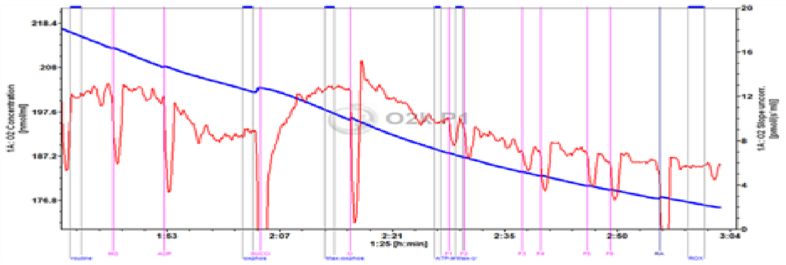

MJD78 JM 0.3

Repeat 1

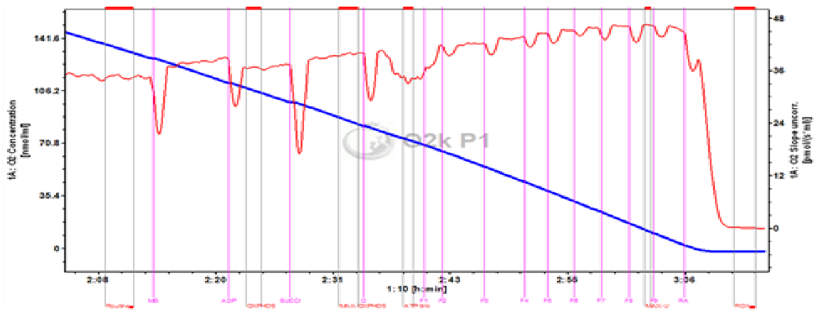

Repeat 2

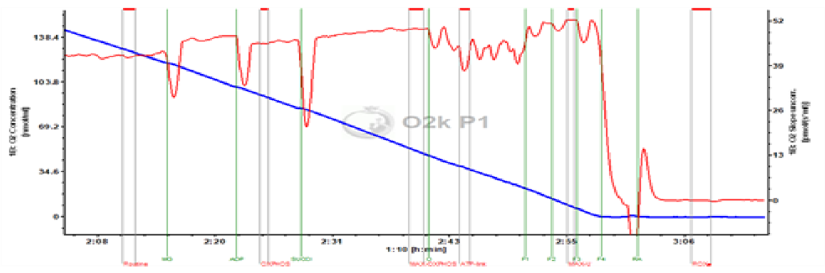

Repeat 3

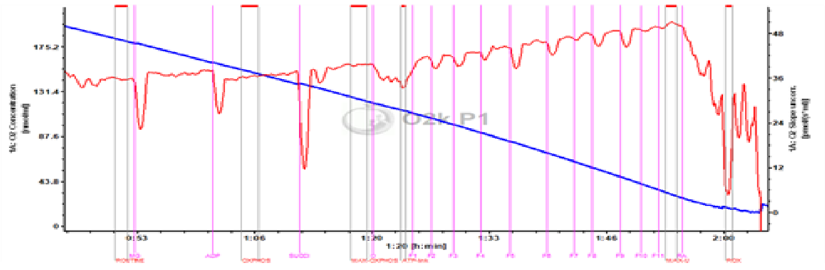

MJD78 JM 1

Repeat 1

Repeat 2

Repeat 3

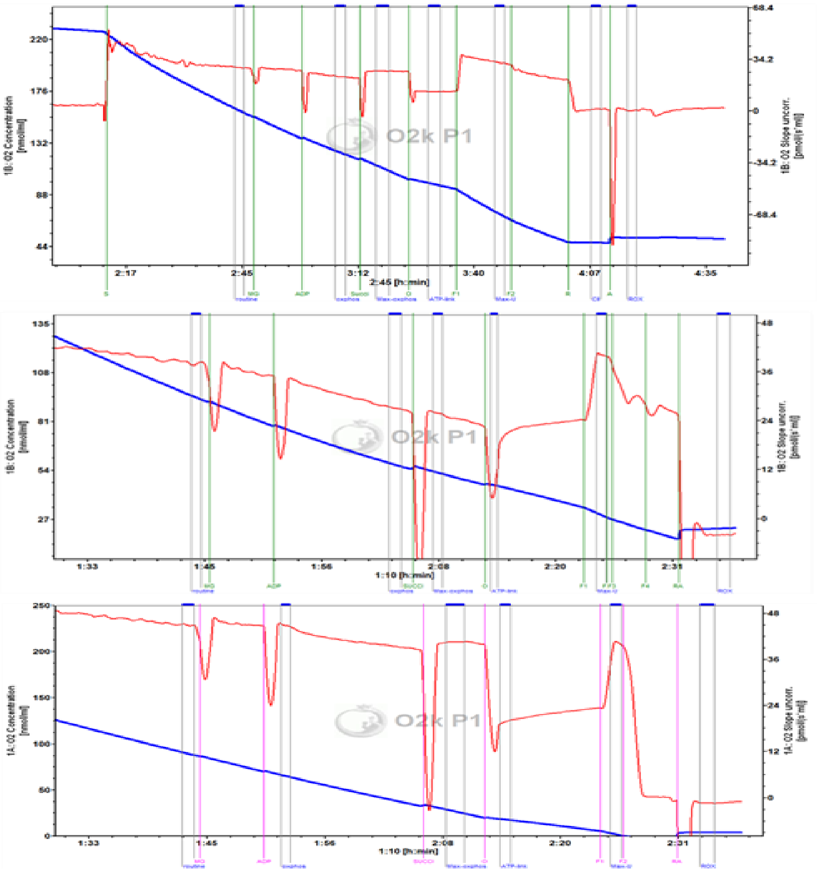

MJD78 JM 5

Repeat 1

Repeat 2

Repeat 3

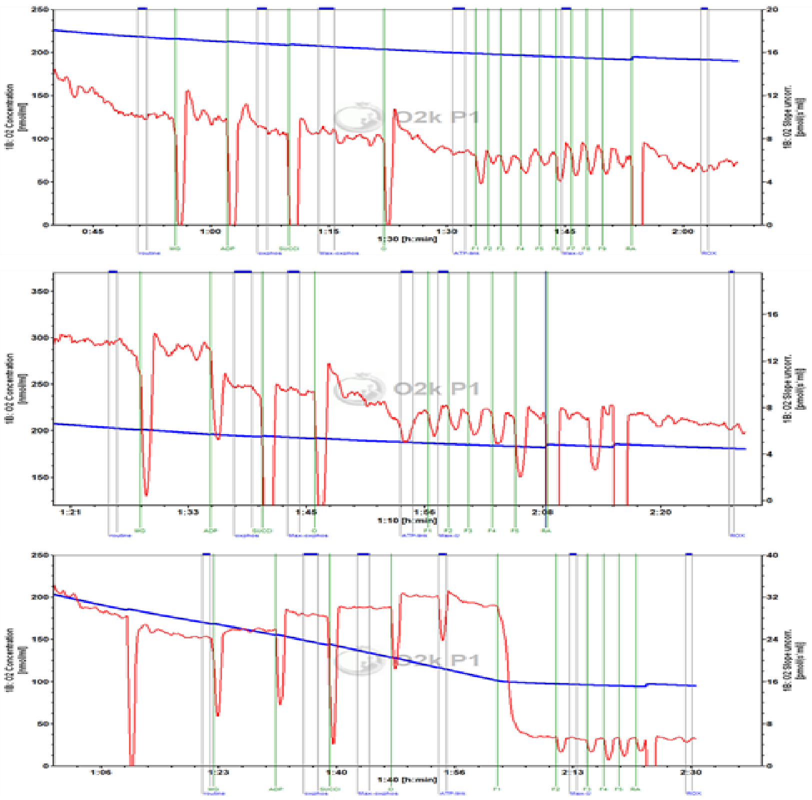

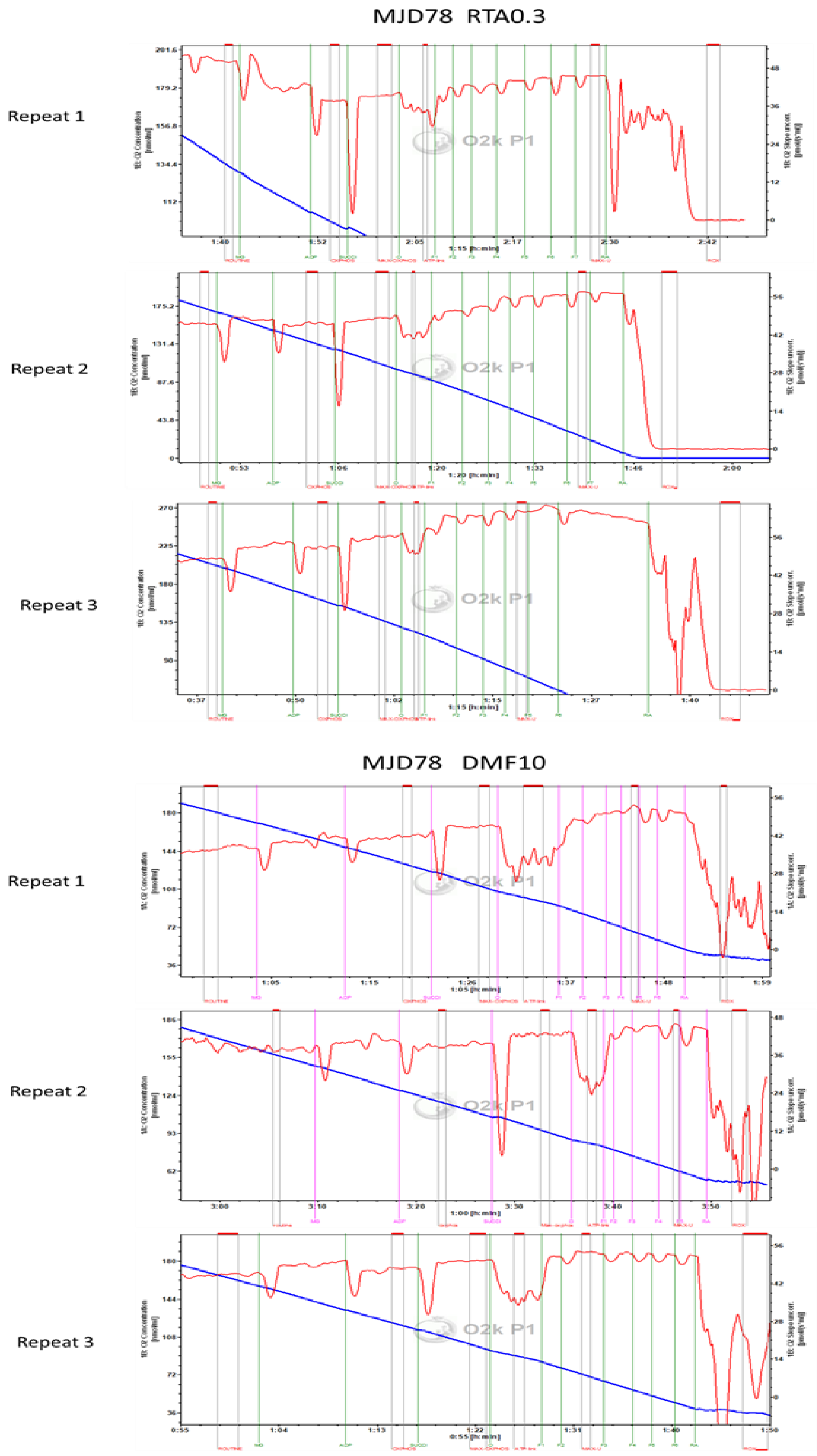

Figure S4. Effects of JM17, RTA-408 and DMF on mitochondrial respiration in MJD78 cells. Cells were treated with or without DMSO vehicle control or 0.3, 1 and 5 μM JM17 or 0.3 μM RTA-408 or 10 μM DMF for 24 hours. The mitochondrial respiration function was measured using OroborosR Oxygraph-2K assay. Blue line was performed to measure decreasing oxygen concentration in the chamber containing living cells. Red line represents the profiles of oxygen flux to allow to conclude the oxygen consumption in respiratory chain. Data are presented at three independent experiments for each group.

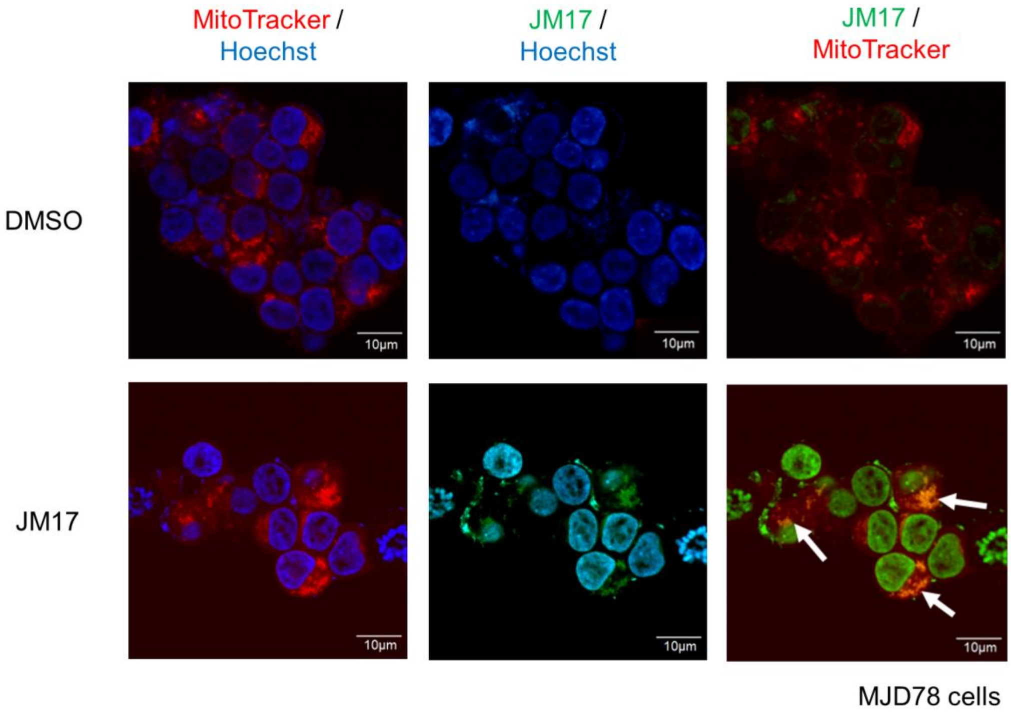

Figure S5. Distribution of JM17 in MJD78 cells. MJD78 cells were treated with or without DMSO vehicle control or 1 μM JM17 for 24 hours before collected and then analyzed by inverted laser scanning Confocal Microscope (Olympus). Arrows show JM17 accumulates in the cellular mitochondria. Scale bar: 10 μm; red fluorescence, MitoTracker Deep Red FM Mitochondrion-selective probes indicator; blue fluorescence, Hoechst 33342 probes nuclear indicator; green fluorescence, belong to JM17 fluorescence.
